# Gene editing therapy for cardiovascular diseases

**DOI:** 10.1002/mco2.639

**Published:** 2024-07-05

**Authors:** Xinyu Wu, Jie Yang, Jiayao Zhang, Yuning Song

**Affiliations:** ^1^ State Key Laboratory for Diagnosis and Treatment of Severe Zoonotic Infectious Diseases Key Laboratory for Zoonosis Research of the Ministry of Education and College of Veterinary Medicine Jilin University Changchun China

**Keywords:** cardiovascular disease, CRISPR/Cas, gene therapy, lipid nanoparticles

## Abstract

The development of gene editing tools has been a significant area of research in the life sciences for nearly 30 years. These tools have been widely utilized in disease detection and mechanism research. In the new century, they have shown potential in addressing various scientific challenges and saving lives through gene editing therapies, particularly in combating cardiovascular disease (CVD). The rapid advancement of gene editing therapies has provided optimism for CVD patients. The progress of gene editing therapy for CVDs is a comprehensive reflection of the practical implementation of gene editing technology in both clinical and basic research settings, as well as the steady advancement of research and treatment of CVDs. This article provides an overview of the commonly utilized DNA‐targeted gene editing tools developed thus far, with a specific focus on the application of these tools, particularly the clustered regularly interspaced short palindromic repeat/CRISPR‐associated genes (Cas) (CRISPR/Cas) system, in CVD gene editing therapy. It also delves into the challenges and limitations of current gene editing therapies, while summarizing ongoing research and clinical trials related to CVD. The aim is to facilitate further exploration by relevant researchers by summarizing the successful applications of gene editing tools in the field of CVD.

## Graphical abstract

Several gene editing systems have been utilized to target crucial genes associated with cardiovascular disease in different animal models through diverse delivery methods, all of which have displayed encouraging outcomes. Recent clinical trials have showcased notable advancements in multiple gene editing therapeutic strategies for cardiovascular disease. Both preclinical and clinical gene editing therapies for cardiovascular diseases establish a strong groundwork for further research and the development of novel therapeutic interventions for cardiovascular diseases

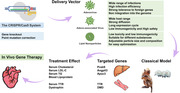

## 1 INTRODUCTION

In 1865, Mendel^1^ proposed the concept of hereditary factors (then known as elementen), which suggested that genetic material controls the inheritance of biological traits. On this basis, Wilhelm Johannsen is credited with proposing the notions of genotype and phenotype in 1909.^2^ Since then, research on gene function and inherited diseases has advanced toward the ultimate goal of gene therapy. The first gene repair in mammalian cells was achieved by Szybalska and Szybalski in 1962,^3^ and in 1990, Ashanti DeSilva became the beneficiary of the world's first licensed gene therapy study.^4^ Gene therapy has evolved and become viable over time and has been used for various systemic disorders, such as cancer, neurodegenerative diseases, cardiovascular diseases (CVDs), and viral infections.^5^, ^6^, ^7^, ^8^

CVDs encompass a variety of heart and vascular conditions, including coronary heart disease, cerebrovascular disease, peripheral artery disease, and congenital heart disease, among others. CVDs pose a significant threat to human health and have emerged as a major global public health concern and social burden.^9^ According to the World Health Organization (WHO), CVD accounts for the majority of deaths caused by noncommunicable diseases, with approximately 17.9 million people affected annually (WHO, 2023). Approximately 85% of deaths related to CVDs are attributed to heart attacks and strokes (WHO, 2021), which are frequently triggered by blockages impeding blood flow to the heart or brain. These blockages are commonly associated with atherosclerosis. Over the past 50 years, there has been remarkable success in reducing cardiovascular mortality in the United States.^10^ However, CVD remains the leading cause of incidence and death.^11^ The increasing medical expenses and reliance on long‐term medication have made CVD a significant public health concern.

On the other hand, extensive research has uncovered a multitude of genetic mutations and risk factors associated with CVD, offering numerous potential targets for therapeutic intervention. These findings provide strong motivation and support for the advancement of gene therapy in the field of CVD. The progress of gene editing therapy for CVDs is a comprehensive reflection of the practical implementation of gene editing technology in both clinical and basic research settings, as well as the steady advancement of research and treatment of CVDs. The continuous efforts of researchers are still required for new pathogenic mechanism research and technological innovation, especially the development of treatment strategies like genome editing.

This review offers an overview of the advancements in gene editing tools for CVD therapy, discussing the challenges and limitations of current gene editing therapies. It also summarizes ongoing research and clinical trials in the field of CVD and provides insights into future directions and prospects in CVD therapy.

## 2 GENE EDITING TECHNIQUES FOR CVDs

Over 400 million people worldwide have been diagnosed with one of around 7000 Mendelian diseases (National Human Genome Research Institute, 2021). These diseases are commonly attributed to a single gene mutation. Gene therapy has emerged as a significant approach to treating genetic diseases in recent years. While gene deletions can be addressed by introducing external genes, the correction of abnormal genes primarily depends on gene editing techniques. In 1985, Smithies et al.^12^ and Thomas and Capecchi^13^ independently developed gene‐targeting technology using homologous recombination and embryonic stem cell techniques pioneered by Martin Evans. However, this technology is known to be labor intensive and time consuming. Often, researchers need to screen a large number of cells to find a successful gene‐targeting event. The development of gene editing technology with the advantages of low cost, easy operation, and high efficiency is of particular significance.

### 2.1 Zinc finger nuclease

In 1996, Chandrasegaran et al.^14^ introduced zinc finger nuclease (ZFN), the first programmable nuclease. The primary purpose of ZFN was to target and cleave specific sites in DNA. ZFN consists of two components. The first component is a zinc finger protein (ZFP) that exhibits high specificity in recognizing triplet bases. Once the modular ZFP is programmed, it can target specific positions in the genomic DNA. The second component is the Fokl endonuclease, responsible for DNA cleavage. By leveraging the cell's inherent DNA damage repair mechanism, modifications can be made to the target gene.

In 2002, Bibikova et al.^15^ successfully knocked out the Yellow gene in Drosophila using ZFN. ZFNs have significantly improved the specificity and accuracy of genome editing compared with gene‐targeting technology. However, practical application faces challenges such as off‐targeting due to the homodimer effect and toxicity of cleaved cells. In 2007, Miller et al.^16^ and Szczepek et al.^17^ developed a variant of FokI with heterodimer activity to reduce off‐target effects. Despite this advancement, the manual design, testing, and screening required for ZFP still pose an obstacle.

### 2.2 Transcription activator‐like effector nucleases

In 2009, Bogdanove and Moscou^18^ and Bonas et al.,^19^ in separate research efforts, pioneered the transcription activator‐like effector nucleases (TALENs) technology. They replaced ZFP with transcription activator‐like effectors (TALEs). Each TALE can bind to a single DNA base, and its modular proteins can bind to specific DNA sequences after programming. The assembly of DNA‐binding repeat arrays has become easier and more efficient due to the development and advancement of various construction methods.^20^

In comparison with ZFN technology, TALENs technology also utilizes proteins for DNA recognition, demonstrating higher efficiency and specificity, as well as being easier to design and implement. TALENs technology remains a significant genetic tool in functional genomics research^20^ and holds a crucial position in mitochondrial gene editing.^21^ Nonetheless, it is essential to acknowledge that TALENs technology is still susceptible to off‐target effects and faces delivery challenges due to its larger molecular structure.

### 2.3 The CRISPR/Cas system

#### 2.3.1 The discovery and development of the CRISPR/Cas systems

The clustered regularly interspaced short palindromic repeat/CRISPR‐associated genes (Cas) (CRISPR/Cas) systems are a remarkable discovery in nature. In 1987, microbiologist Yoshizumi Ishino unexpectedly discovered abnormal repetitive sequences in Escherichia coli.^22^ Later, Mojica et al.^23^ discovered this specific sequence in various microorganisms and paid continuous attention to it. In 2002, Jansen et al.,^24^ upon Mojica's suggestion, defined CRISPR and discovered the presence of Cas near the CRISPR sequence. At that time, the functionality of the CRISPR system had not been confirmed.

Until 2005, three independent research teams, led by Mojica et al.,^25^ Vergnaud et al.,^26^ and Bolotin et al.,^27^ reported that the interval between CRISPR repeat sequences originated from phage DNA and extracellular DNA, such as plasmids. Later, Koonin et al.^28^ proposed that Cas might function as a prokaryotic defense system through the RNAi mechanism. He also suggested the hypothesis of CRISPR/Cas as an acquired immune system. In 2007, Horvath's team^29^ made a significant discovery that bacteria integrated new spacer sequences from phage genomes after being attacked. The removal or addition of specific interval sequences was found to alter the phage resistance phenotype of cells.^29^ This study provided the first experimental evidence of CRISPR/Cas as a bacterial‐acquired immune system and garnered significant attention from researchers in this field.

In 2008, van der Oost et al.^30^ furthered the research by discovering that mature crRNA (CRISPR RNA) can guide Cas protein complexes to interfere with virus proliferation, with the assistance of helicase. They also proposed the hypothesis that CRISPR targets DNA. It has been confirmed in a study conducted by Marraffini and Sontheimer.^31^ They discovered that CRISPR targets DNA in Staphylococcus epidermidis and identified its ability to cleave or correct the genome in eukaryotic cells.^31^ In 2009, the Mojica team^32^ identified proto‐spacer adjacent motifs (PAMs) to determine the targets of the prokaryotic CRISPR defense system. In 2010, Moineau's team^33^ demonstrated that the CRISPR/Cas system of Streptococcus thermophilus cleaves plasmid and phage double‐stranded DNA at three nucleotides upstream of the PAM sequence. These studies collectively show that Cas is guided by crRNA and induces double‐stranded breaks in DNA.

In 2011, the Charpentier research team^34^ discovered a small RNA known as trans‐activating CRISPR RNA (tracrRNA). The researchers highlighted that tracrRNA plays a vital role in guiding the maturation of crRNAs through the actions of two important proteins: the widely conserved endogenous RNase III and the CRISPR‐associated Csn1 protein.^34^ These proteins are essential for safeguarding Streptococcus pyogenes from invasion by phage DNA. Subsequently, Charpentier et al.^35^ discovered that tracrRNA not only plays a role in the processing of crRNA but also forms a distinct secondary structure with crRNA, which facilitates DNA splicing. Moreover, they demonstrated that by combining tracrRNA and crRNA into a single RNA chimera while maintaining the secondary structure, DNA splicing can still be guided. These findings shed light on the vast potential of the CRISPR/Cas system for gene editing through RNA guidance.

In 2011, the team led by Siksnys et al.^36^ introduced the CRISPR/Cas9 system of Streptococcus thermophilus into Escherichia coli. This system offered protection against heterologous invasion from plasmid transformation and phage infection. Additionally, in 2012, the same team validated the in vitro activity of the CRISPR/Cas9 system complex of Streptococcus thermophilus,^37^ which aligned with the findings of a contemporaneous study by Charpentier et al.^35^ In early 2013, two independent teams, Zhang et al.^38^ and Church et al.,^39^ successfully achieved precise cleavage of endogenous genomes within mammalian cells. They were able to introduce multiple guide RNAs (gRNAs) simultaneously, allowing for the editing of several loci within the genome. These researches showcased the CRISPR/Cas system's easy programmability and wide applicability.

**FIGURE 1:**
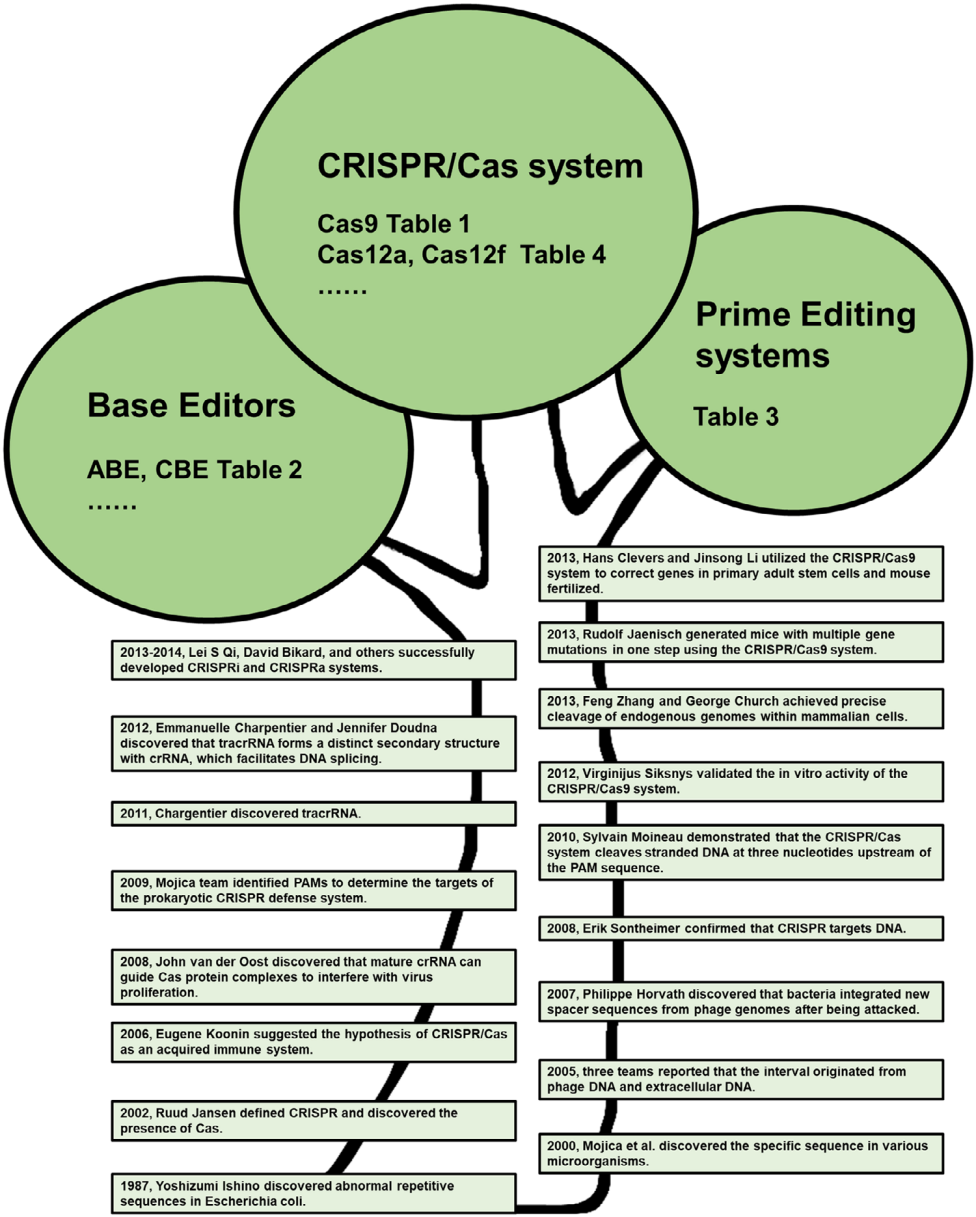
The discovery and development of the CRISPR/Cas system. The CRISPR system has rapidly expanded since its initial discovery, emerging as a crucial tool for gene editing therapies. *Abbreviations*: ABE, Adenine Base Editor; CBE, Cytidine Base Editor; CRISPR, clustered regularly interspaced short palindromic repeats; Cas, CRISPR‐associated.

The CRISPR/Cas system was soon utilized for manipulating gene expression. Researchers such as Qi, Bikard, and others^40^, ^41^, ^42^ successfully developed CRISPR interference (CRISPRi) and CRISPR activation (CRISPRa) systems by attaching catalytic inactivated Cas9 (dCas9) to transcription inhibitors or activators. These systems enable the regulation of gene expression within a 1000‐fold range and offer valuable insights for screening and identifying growth essential genes, tumor suppressors, differentiation regulatory factors, and mapping complex pathways. In May 2013, Jaenisch's team^43^ successfully generated mice with multiple gene mutations in one step using the CRISPR/Cas9 system. This method eliminated the need for the laborious process of modifying embryonic stem cells and cultivating several generations of mice. As a result, it significantly accelerated functional research on multiple genes in vivo. Since then, the CRISPR/Cas system has been widely utilized in genome engineering for various organisms. It has achieved successful gene editing in animals like fruit flies, rats, pigs, and monkeys, as well as in plants such as rice, corn, and soybeans. In December 2013, two research groups, Clevers et al.^44^ and Li et al.,^45^ utilized the CRISPR/Cas9 system to correct genes in primary adult stem cells from patients with monogenic genetic defects and mouse‐fertilized eggs with dominant mutations. This marked the first step toward gene therapy for genetic diseases based on CRISPR/Cas system. From 2012 to 2013, CRISPR/Cas technology made significant advancements, progressing from in vitro reconstruction to gene editing of human cells. This breakthrough has led to a surge in research on CRISPR/Cas technology, which continues to this day. The discovery and development of the CRISPR/Cas system have been accompanied by the unwavering enthusiasm of countless researchers, heralding a new era of gene editing technology and gene therapy for diseases, as depicted in Figure 1.

#### 2.3.2 CRISPR/Cas9 system

Since its initial discovery, CRISPR/Cas system has primarily been used for editing DNA. Prokaryotic cells can combat foreign invaders by memorizing and cutting their nucleic acid.^29^ Different bacteria and archaea use varying implementation methods, which can be categorized based on the Cas gene classification and the nature of the interference complex.^46^ Class I systems encompass types I, III, and IV, in which crRNA forms complexes with multiple proteins and binds to target DNA, while Class II systems include types II, V, and VI, in which crRNA forms complexes with a single protein.^47^ Currently, the most widely used gene editing system is CRISPR/Cas9, which is part of the Type II systems. Other commonly used editing tools include Cas12a and Cas12f, which belong to the Type V systems.

The CRISPR/Cas9 system consists of two main components: gRNA and Cas9 protein. gRNA is a small RNA molecule made up of crRNA and tracrRNA, which guides the binding to the target DNA site. The Cas9 protein is primarily composed of the recognition lobe (REC) and the nuclease lobe (NUC). The NUC lobe is made up of the RuvC, HNH, Wedge (WED), and PAM‐interacting (PI) domains. The HNH domain is responsible for cleaving complementary chains, while the RuvC domain is responsible for cleaving noncomplementary chains. Once gRNA binds to its target site, it recruits Cas9 protein to cleave the DNA at that site. Different bacteria or archaea have different Cas9 proteins, such as SpCas9, SaCas9, FNCas9, and others. Currently, SpCas9 and its variants are extensively utilized as the Cas9 protein in numerous in vitro and in vivo experiments. In the realm of gene therapy, SaCas9 has emerged as a promising candidate owing to its compact size and convenient integration into delivery systems.

The CRISPR/Cas9 system is commonly used for gene editing by causing targeted double‐stranded DNA breaks (DSBs).^48^ These breaks can lead to a variety of human illnesses in mammalian cells, and the cells have developed mature repair mechanisms, including nonhomologous end joining (NHEJ) and homology‐directed recombination.^49^ The NHEJ repair mechanism is not always accurate in repairing DSBs in DNA. This inaccuracy can lead to the loss or addition of DNA sequences, causing frameshift mutations. CRISPR/Cas9 system uses the NHEJ repair mechanism to induce frameshift alterations or mutations in the target gene resulting in gene silencing.^50^ This approach can be used for gene therapy to control gene expression.

The gene‐editing potential of the CRISPR/Cas9 system has been recognized since 2012.^35^ However, it still faces certain challenges that require attention. These include editing efficiency, off‐targeting, PAM limitation, inefficient homologous recombination, and the need for miniaturization of tools. These issues need to be resolved urgently. Over the last decade, the CRISPR/Cas9 system has garnered significant attention and funding, leading to advancements in enhancing its usability, efficiency, and safety. As shown in Table 1, researchers have proposed numerous solutions to address the limitations and challenges and have consistently pursued iterative improvements. They are actively exploring and discovering novel Cas9 proteins in different bacteria or archaea species to broaden the available options. Simultaneously, researchers are also focusing on enhancing the classical Cas9 proteins by reducing their size, improving efficiency, and ensuring safety. This is achieved through structural modifications such as deletion and optimization, as well as introducing amino acid mutations at specific sites. In 2024, Doudna's team^51^ utilized single‐particle cryo‐electron microscopy structural analysis and biochemical experiments to demonstrate that mutating a specific point in the WED functional domain of iGeoCas9 improves its DNA binding capacity and speeds up DNA deconjugation. This enhancement ultimately increases the efficiency of gene editing by iGeoCas9 in mammalian cells. These advancements are the result of the tireless efforts of countless scientists in both basic and applied research, culminating in a breakthrough in gene therapy.

**TABLE 1:** The progress of the clustered regularly interspaced short palindromic repeat/CRISPR‐associated genes systems.

Name	Mutation(s)	PAM	Size	References
SpCas9		NGG	1368	^38^
SpCas9‐ VQR, ‐EQR, ‐VRER	D1135V/R1335Q/T1337R, D1135E/R1335Q/T1337R, or D1135V/G1218R/R1335E/T13 37R	NGAN, NGNG, NGAG, or NGCG	1368	^52^
xCas9‐3.7	A262T/R324L/S409I/E480K/E543D/M694I/E1219V	NG, NNG, GAA, GAT, and CAA	1368	^53^
SpCas9‐NG	R1335V/L1111R/D1135V/G1218R/E1219F/A1322R/T1337R	NG	1368	^54^
ScCas9		NNG	1375	^55^
ScCas9++	10‐aa region swap T1227K	NNG	1375	^56^
StCas9		NNAGAAW	1121	^57^
NmeCas9		NNNNGATT	1082	^58^
Nme2Cas9		NNNNCC	1082	^59^
Nme3Cas9		NNNNCAAA	1081	^59^
SaCas9		NNGRRT	1053	^60^
SaCas9 KKH	E782K/N968K/R1015H	NNNRRT	1053	^61^
SaCas9 v42	13‐aa region swap	NNVRRN	1053	^62^
SaCas9 V17‐L	13‐aa region swap I991L	NNVRRN	1053	^62^
SaCas9 v21‐R, v21‐L	13‐aa region swap I991R, I991L	NNGACT, NNGATG, NNGATT, NNGGCT, NNGGTG, and NNGGTT	1053	^62^
Sa‐SchCas9	SchCas9 PI replaced SaCas9 PI	NNGR	1055	^63^
CjCas9		NNNRYAC	984	^64^
GeoCas9		NNNNCRAA	1087	^65^
FnCas9		NGG	1629	^66^
FnCas9 RHA	E1369R/E1449H/R1556A	YG	1629	^67^
SpG	D1135L/S1136W/G1218K/E1219Q/R1335Q/T1337R	NGN	1368	^68^
SpRY	A61R/L1111R/D1135L/S1136W/G1218K/E1219Q/N1317R/A1322R/R1333P/R1335Q/T1337R	NRN>NYN	1368	^68^
eNme2‐T.1		NNNNTN	1082	^69^
eNme2‐T.2		NNNNTN	1082	^69^
eNme2‐C		NNNNCN	1082	^69^
eNme2‐C.NR		NNNNCN	1082	^69^
vCas9	S55R/R976A/K1003A/T1314R	NGG	1368	^70^
SpRYc	Recombine the PAM‐interacting domain of SpRY with the N‐terminus of Sc++.	NNN	1368	^71^
iGeoCas9	GeoCas9 E149G/T182I/N206D/P466Q/Q817R/E843K/E884G/K908I	NNNNCNNN	1087	^51^

Abbreviations: aa, amino acid; PAM, proto‐spacer adjacent motif.

### 2.4 Base editor

The function of the CRISPR/Cas9 system is determined by its structure. The HNH and RuvC domains play a crucial role in cleaving the target site, thus determining the efficiency and outcomes of the entire system. Researchers have discovered additional applications for the system while striving to enhance its efficiency. The nCas9 and dCas9 systems were created by modifying the protein structure and deactivating one or both of the HNH and RuvC domains. This modification enables them to generate single‐strand breaks or even lose the capability to cut DNA at the desired location. Consequently, this advancement has paved the way for various applications such as precise gene editing, base editing, gene composition imaging, and epigenetic regulation.

Indeed, a significant number of genetic diseases are known to be caused by mutations in DNA bases. However, conventional gene editing tools face challenges in achieving precise modifications to DNA, particularly in the case of point mutations. These tools often struggle to achieve the intended modifications accurately. To achieve targeted gene editing that converts cytidine to uridine (C to T or G to A), Liu et al.^72^ combined Cas9 technology with cytidine deaminase to obtain Cytidine Base Editor (CBE) in 2016. Under the guidance of sgRNA, rAPOBEC1, a cytosine deaminase derived from rats, binds to the designated site and converts cytosine (C) into uracil (U) within the editing window. Simultaneously, nCas9 is used to cut the nonedited DNA strand, and guanine (G) is replaced with adenine (A) through mismatch repair (MMR) to pair with U. Eventually, during DNA replication, U is transformed into thymine (T) to achieve base substitution. Additionally, uracil DNA glycosylase inhibitor (UGI) is included in the fusion protein to inhibit the host's intracellular uracil glycosylase activity. This prevents the modified U from being repaired through base‐excision repair (BER) by the host, thereby improving the editing efficiency. In the same year, Kondo's team^73^ utilized PmCDA1, a cytosine deaminase found in sea lamprey, to create a base editing system known as targeted‐AID. Additionally, Chang's team^74^ developed the targeted AID‐mediated mutagenesis (TAM) system using human AID, while Bassik's laboratory^75^ developed the CRISPR‐X system, which recruits human AID deaminase through MS2. The binding of cytidine deaminase to Cas9 marks the beginning of a significant development in the field. This resulted in the development of base editors, which have become a significant tool in gene therapy. Moreover, this also opens up possibilities for exploring even more fascinating combinations in the future.

In 2017, Liu's team^76^ achieved the Adenine Base Editor (ABE) system after seven rounds of evolution and screening to obtain ecTadA, an adenine deaminase that can be used to enable base editing. This system efficiently and specifically replaces the A base with the G base (A to G or T to C).^76^ The ecTadA could convert adenine (A) into inosine (I) within a specific editing window when it binds to a designated position. Inosine (I) possesses a structure that closely resembles guanine (G), causing the cell to read and replicate it as G. The base editing system improves the efficiency of preparing cellular and animal models of single‐base mutations, making it a valuable tool for studying the pathogenesis and gene therapy of genetic diseases caused by single‐gene single‐point mutations. The CBE and ABE systems have been able to successfully correct 30% of identified human pathogenic variations.^77^

Similar to the Cas9 system, the Base Editor (BE) system encounters challenges with editing efficiency, off‐target effects, and size. Furthermore, it needs to tackle other forms of base substitutions, determine the position and size of the editing window, handle specific challenging sites such as GC, and perform more intricate tasks like multiple base substitutions at multiple sites. As indicated in Table 2, numerous studies have been conducted in this area. On one hand, researchers are actively screening novel functional proteins, while on the other hand, they are enhancing existing base editing systems. This involves optimizing protein structures and codons, adjusting linker sequences between functional elements, as well as determining the positions and quantities of functional elements. These efforts have significantly enhanced the efficiency of base editing and minimized the risk of off‐target effects. Liu's team^78^ developed a series of highly selective CBE variants (CBE6a‐CBE6d) evolved from the dual base editor TadDE by a phage‐assisted evolution approach, which exhibit highly efficient C‐G to T‐A editing activity and little to no A‐T to G‐C off‐target editing activity. Scientists have fused base editors to enable two‐base gene editing, allowing for the replacement of adjacent CA bases.^79^, ^80^, ^81^, ^82^ The CGBE,^83^ GBE,^84^ and gGBE^85^ systems are effective in reversing C‐G bases (C‐to‐G/C‐to‐A) at specific target sites in mammalian cells, thereby enhancing the overall base editing systems. The development of base editors such as AYBE,^86^ AXBE, and ACBE^87^ further enhanced adenine reversal and enabled editing of A to Y (Y = C or T) up to 73%. And, several novel base editors that do not rely on deaminase are in development. Bi's team^88^ developed deaminase‐free (DAF) novel base editors, DAF‐CBE and DAF‐TBE, through the utilization of two mutants derived from the directed evolution of human uracil glycosylase (UNG). The development of various base‐editing tools holds significant importance in creating human genetic disease models and advancing the field of gene therapy.

**TABLE 2:** The progress of the base editors.

Name	Architecture	Editing window	Editing efficiency	Note	References
BE3	rAPOBEC1‐nCas9‐UGI	C4–C8	Up to 37% in human cells	Prefers TC motifs	^72^
Target‐AID	nCas9‐PmCDA1‐UGI	C2–C5	Up to 59.9% in CHO cells		^73^
TAM	dCas9‐hAID(P182X)	C4–C8	N/A	Used for random mutagenesis	^74^
CRISPR‐X	dCas9‐MS2‐hAID	C‐50–C50	N/A	Used for random mutagenesis	^75^
YE1‐BE3	rAPOBEC1 (W90Y/R126E) nCas9‐UGI	C5–C7	Comparable to BE3		^89^
YE2‐BE3	rAPOBEC1 (W90Y/R132E) nCas9‐UGI	C5–C6	Comparable to BE3		^89^
YEE‐BE3	rAPOBEC1 (W90Y/R126E/R132E) nCas9‐UGI	C5–C6	Comparable to BE3		^89^
SECURE‐BE3	rAPOBEC1(R33A/K34A)‐nCas9‐UGI	C5–C7	Comparable to BE3	Substantially decreased the number of RNA edits	^90^
FNLS‐BE3	rAPOBEC1‐nCas9‐UGI	C3–C8	80–95% in NIH/3T3 cells		^91^
eA3A‐BE3	hAPOBEC3A (N57G)‐nCas9‐UGI	C4–C8	22.5% in HEK293T cells	Prefers TCR motifs	^92^
hA3A‐BE3	hAPOBEC3A (Y130F)‐nCas9‐UGI	C3–C8	Higher editing efficiencies than BE3 (median ∼2.3 fold) in HEK293T cells	Mediated efficient base editing in regions with high methylation levels and GpC dinucleotide content	^93^
hA3A‐BE3	hAPOBEC3A (Y132D)‐nCas9‐UGI	C3–C7	Higher editing efficiencies than BE3 (median ∼1.2–1.9 fold) in HEK293T cells	Mediated efficient base editing in regions with high methylation levels and GpC dinucleotide content	^93^
eA3G‐BE	hA3Gctd‐nCas9‐UGI‐UGI	C4–C10	18.3−58.0% in HEK293T cells; 54.5–92.2% in rabbit embryos	Prefers CCC>CCC>CC motifs	^94^
oA3G‐BE3	hA3G(R24A/W94L/Y124A/W127L/P200K)‐nCas9‐UGI	C3–C7	Up to 41% in HEK293T cells	Prefers CCC and CC motifs	^95^
eAID‐BE4max	hAID(K10E/T82I/E156G/P182X)‐nCas9‐UGI‐UGI	C1–C11	Up to 78.31 ± 3.87% in rabbit embryos	With significantly improved base‐editing efficiency in GC contexts	^96^
BE‐PLUS	SunTag‐nCas9‐scFv‐rAPOBEC1‐UGI	C4–C14	Induced far more C‐to‐T conversions at positions 9−16 as compared with BE3	With increased editing window and enhanced fidelity	^97^
BE4	rAPOBEC1‐nCas9‐UGI‐UGI	C3–C8	Observed an average increase in C‐to‐T editing efficiencies of 1.5 ± 0.3‐fold relative to BE3		^98^
BE4‐Gam	Gam‐ rAPOBEC1‐nCas9‐UGI‐UGI	C3–C8	Observed no apparent decreases in C‐to‐T editing efficiencies for BE4‐Gam relative to BE4	Exhibited greatly decreased indel frequencies; with an average decrease in non‐T product formation	^98^
BE4‐Max	rAPOBEC1‐nCas9‐UGI‐UGI	C4–C8	Averaged 89 ± 0.9% in HEK293T cells		^99^
AncBE4‐Max	rAPOBEC1 (Anc689)‐nCas9‐UGI‐UGI	C4–C8	Averaged 90 ± 1.5% in HEK293T cells		^99^
evoBE4max	rAPOBEC1‐nCas9‐UGI‐UGI	C3–C8	25 ± 9.8% in HEK293T cells	More efficient at editing GC	^100^
evoFERNY‐BE4max	rAPOBEC1‐nCas9‐UGI‐UGI	C3–C8	24 ± 13% in HEK293T cells	29% smaller size compared with APOBEC1	^100^
CBE6	CBE6a (TadDE N46I Y73P) CBE6b (TadDE N46V Y73P) CBE6c (TadDE N46L Y73P) CBE6d (TadDE N46C Y73P)	C4–C8	Exceeding BE4max and previous TadCBEs in human cells	Without creating unwanted missense mutations from residual A•T‐to‐G•C editing	^78^
ABE7.10	ecTadA‐TadA*7.10‐nCas9	A4–A7	34−68% in HEK293T cells		^76^
ABEmax	ecTadA‐TadA*7.10‐nCas9	A4–A7	Induced 46 ± 0.55% (unsorted) and 52 ± 5.2% (sorted) editing in HEK293T cells		^99^
ABE7.10 F148A	ecTadA(F148A)‐TadA*7.10(F148A)nCas9	A5–A6	Similar to ABE7.10		^101^
SECURE‐ABE	TadA*7.10(K20A/R21A)‐nCas9	A4–A7	7.9−70.9% in HEK293T cells		^102^
SECURE‐ABE	TadA*7.10(V82G)‐nCas9	A4–A7	10.6−59.4% in HEK293T cells		^102^
ABE8e	TadA*8e‐nCas9	A4–A8	18−86% in HEK293T cells		^103^
ABE9	TadA*8e(N108Q/L145T)‐nCas9	A5–A6	36.08–62.41% in rat embryos		^104^
ABE8r	TadA*8r‐nCas9	A2–A9	65.1 ± 11.8% in HEK293T cells	High activity at RA and PAM‐distal A sites	^105^
CGBE1	eUNG‐rAPOBEC1(R33A)‐nCas9	C5–C7	41.7–71.5% in HEK293T cells	C‐to‐G	^83^
GBE	rAPOBEC1‐nCas9‐UNG	C6	5.3–53.0% in HEK293T cells	C‐to‐G	^84^
CGBE(XRCC1)	rAPOBEC1‐nCas9‐XRRCC1	C5–C6	15.4 ± 7% in human cells	Within a three nucleotide target window and WCW, ACC, and GCT target sequence contexts	^106^
ABE‐P48R	TadA*7.10(P48R)‐nCas9	C5–C7	N/A	TC‐to‐TG	^107^
Td‐CGBE	TadA*8e(N46L)‐nCas9	C5–C6	Up to 72.8% in HEK293T cells	C‐to‐G	^108^
GBE2.0	rAPOBEC1(R33A)‐nCas9‐Rad51‐Ung1	C6	8.70–72.1% in HEK293T cells	C‐to‐G	^109^
gGBE	nCas9‐MPG	G6–G11	Up to 46.3% in N2a cells	G‐to‐T or G‐to‐C (i.e. G‐to‐Y)	^85^
AYBEv3	TadA*8e‐nCas9‐MPG	A5–A9	8–72% in HEK293T cells	A‐to‐C or A‐to‐T (i.e. A‐to‐Y)	^86^
AXBEv2	TadA*8e‐nCas9‐ mAAG(R165E/Y179F)	A2–A12	A‐to‐Y transversion efficiency (52.8%) in HEK293T cells	A‐to‐C or A‐to‐T (i.e. A‐to‐Y)	^87^
ACBE‐Q	TadA*8e(Ce07) (N108Q)‐nCas9‐ mAAG(R165E/Y179F)	A4–A6	Averaging 27% (up to 45%) in HEK293T cells	A‐to‐C	^87^
DAF‐CBE	CDG4‐nCas9	C2–C6	With an average editing efficiency of 20.7%, in which the editing efficiencies of C‐to‐G, C‐to‐T, and C‐to‐A were 7.3, 10.8, and 2.6%, in HEK293T cells	C‐to‐G, C‐to‐T and C‐to‐A	^88^
DAF‐TBE	TDG3‐nCas9	T2–T6	With an average editing efficiency of 22.5%, in which the editing efficiencies of T‐to‐G, T‐to‐C, and T‐to‐A were 13.4, 6.4, and 2.7%, in HEK293T cells	T‐to‐G, T‐to‐C and T‐to‐A	^88^

### 2.5 Prime editing system

Although the BE system has successfully corrected many harmful mutations, there are still genetic challenges that need to be addressed, such as the precise insertion and deletion of genes. In 2019, Liu's team^110^ introduced the Prime Editing (PE) system, which significantly expanded the application range of base editors by combining nCas9 with reverse transcriptase (RT) and adaptively updated and optimized gRNA. The PE system is dependent on PE guide RNA (pegRNA). The 3′ end of pegRNA contains a prime‐binding site that pairs with the target DNA strand to be cleaved. It also has a RT template for reverse transcription by RT at the 3′ end of the broken DNA single strand, which generates the edited sequence. Subsequently, the 5′ end nuclease cuts the unedited 5′ ssDNA, resulting in DNA editing. To enhance the editing efficiency, sgRNA matching the edited strand is utilized to create a second nick in the unedited complementary strand. This encourages DNA repair to preferentially replace the unedited strand. The PE method can achieve more precise and complex gene editing through point mutation or insertion–deletion mutation of reverse transcription templates.

However, the editing efficiency of the PE system is still low, and the design of pegRNA sequences is relatively complex. As the PE system is currently one of the most popular and promising gene editing systems, researchers are actively searching for optimization solutions. Table 3 presents the progressive improvement in editing efficiency and editing scope of the PE system, along with different editing strategies developed to address various usage scenarios such as multifocus mutation, large segment deletion, replacement, and replication.

**TABLE 3:** The progress of the prime editing systems.

Name	Architecture	Note	References
PE1	nCas9(H840A)‐MMLV RT; pegRNA		^110^
PE2	nCas9(H840A)‐MMLV RT mutant*; pegRNA		^110^
PE3	nCas9(H840A)‐MMLV RT mutant*; pegRNA; nsgRNA		^110^
PEmax	nCas9(R221K/N394K/H840A)‐MMLV RT mutant*; pegRNA	Can improve nuclear localization, reverse transcriptase expression, and Cas9 nuclease activity	^111^
hyPE2	nCas9(H840A)‐Rad51 DBD‐MMLV RT mutant*; pegRNA		^136^
PE2ΔRnh	nCas9(H840A)‐MMLV RT mutant* ∆RNaseH; pegRNA		^130^
PE4	PE2; MLH1dn	Can transiently inhibit MMR and enhance prime editing efficiency	^111^
PE5	PE3; MLH1dn	Can transiently inhibit MMR and enhance prime editing efficiency	^111^
PE6a	nCas9(R221K/N394K/H840A)‐evo‐ Ec48rt(E60K/K87E/E165D/D243N, R267I/E279K/K318E/K343N); pegRNA; nsgRNA		^129^
PE6b	nCas9(R221K/N394K/H840A)‐evo‐ Tf1rt(P70T/G72V/S87G/M102I/K106R/K118R/I128V/L158Q/F269L/A363V/K413E/S492N); pegRNA; nsgRNA		^129^
PE6c	nCas9(R221K/N394K/H840A)‐evo‐ Tf1rt(P70T/G72V/S87G/M102I/K106R/K118R/I128V/L158Q/F269L/A363V/K413E/S492N/K118R/S188K/I260L/S297Q/R288Q); pegRNA; nsgRNA		^129^
PE6d	nCas9(R221K/N394K/H840A)‐M‐MLVRTDRNAseHrt(T128N/V223Y/D200C); pegRNA; nsgRNA		^129^
PE6e	nCas9(H840A/K918A/K775R)‐M‐MLVRTDRNAseHrt	Can better enhance mammalian PE efficiency	^129^
PE6f	nCas9(H840A/E471K/H99R/I632V/H721Y/D645N/K918A)‐M‐MLVRTDRNAseHrt	Can better enhance mammalian PE efficiency	^129^
PE6g	nCas9(H840A/E471K/H99R/I632V/H721Y/R654C/D645N)‐M‐MLVRTDRNAseHrt	Can better enhance mammalian PE efficiency	^129^
Dual‐pegRNA	Using two pegRNAs in trans encoding the same edits		^137^
HOPE	Using paired pegRNAs encoding the same edits in both sense and antisense DNA strands		^138^
PRIME‐Del	Using a pair of pegRNAs that target opposite DNA strands, programming not only the sites that are nicked but also the outcome of the repair		^119^
PEDAR	Combining PE‐Cas9 with two pegRNAs targeting complementary DNA strands		^121^
PETI	Programmable chromosomal translocation and inversion using PE2 nuclease and paired pegRNA		^122^
TD‐PE	The RT template of each extension is designed homologous to the target region of the other sgRNA to promote the reannealing of the edited DNA strands and the duplication of the fragment in between.		^123^
TwinPE	Can precisely insert or delete hundreds of base pairs of DNA and can be used in tandem with recombinases to achieve gene‐sized (5 kb) insertions and inversions.		^120^
PASTE	Using a CRISPR–Cas9 nickase fused to both a reverse transcriptase and serine integrase for targeted genomic recruitment and integration of desired payloads		^124^
CPE	Using the smaller Cas12a protein to develop CPE systems	Can preferentially recognize T‐rich genomic regions	^131^
PE7	Fused to the RNA‐binding, N‐terminal domain of La		^132^

*Note*: MMLV RT mutant*, MMLV RT((D200N/T306K/W313F/T330P/L603W).

In 2021, Liu's group^111^ discovered that DNA MMR significantly inhibits the editing activity of PE. To overcome this limitation, the researchers cotransfected the engineered repressor protein MLH1dn along with the previous generation of the PE system.^111^ This strategy aimed to enhance the gene editing efficiency of the PE system by transiently inhibiting MMR. Furthermore, the researchers generated the PEmax architecture by optimizing the RT structure and amino acid sequence within PE, resulting in a substantial improvement. Subsequently, several independent research teams concentrated on enhancing the PE system by modifying pegRNAs. Many programs aimed at protecting the 3′ end of pegRNA have shown excellent results.^112^, ^113^, ^114^, ^115^, ^116^ In addition, two independent research groups, Yang et al.^117^ and Chen et al.,^118^ discovered that introducing synonymous mutations at specific positions in the RT template of pegRNA can greatly enhance the base editing efficiency of PE. However, further exploration is required to determine the ideal design pattern for synonymous mutations to evade MMR.

Multiple research groups have proposed dual pegRNA strategies with varying details.^119^, ^120^, ^121^ These strategies aim to enhance the scope and utility of PE editing by employing two pegRNAs to simultaneously edit two DNA strands. Kim's team^122^ developed prime editor nuclease‐mediated translocation and inversion (PETI), which efficiently generates chromosomal translocations and inversions in a programmable manner with a comparable efficiency to that of Cas9. Yao's team^123^ developed a strategy, tandem duplication via PE (TD‐PE), for creating robust and precise in situ tandem duplications of genomic fragments ranging from ∼50 bp to ∼10 kb. However, the current approach still faces challenges in effectively inserting large fragments of DNA. To address this issue, researchers have introduced serine integrase, which enables targeted recombination between the attB element in the receptor genome and the attP element in the donor DNA. The process begins by preloading the att site into the genome using the PE system. Subsequently, the donor DNA, which contains complementary att sites, is inserted into the preinstalled sites using serine integrase. Two separate groups, David R. Liu and Jonathan S. Gootenberg, developed the twinPE and PASTE systems, respectively, based on this concept.^120^, ^124^ These systems successfully allowed for targeted insertions, deletions, and inversions of DNA sequences that are larger than 5 kb in size. This significant achievement greatly enhances the potential of primer editing and targeted gene integration in mammalian cells and lays the foundation for potential treatments of genetic diseases that involve large segments of genetic abnormalities.

In 2023, the PE5 system^111^ was successfully applied in gene therapy for sickle cell disease (SCD) mouse models.^125^, ^126^ Shortly after, Liu's research group^127^, ^128^ utilized phage‐assisted continuous and noncontinuous evolution (PACE and PANCE, respectively) technology to develop a more compact and efficient PE6a‐g system. PE6a‐b was successfully obtained by the researchers using evo‐Ec48 and evo‐Tf1 RT derivatives. This was achieved through screening 59 RT enzymes, engineered mutations, and PANCE technology. Subsequently, PE6c‐d was obtained by combining protein engineering and PACE technology based on PE6b and PE2. Finally, the researchers screened and identified single mutants of Cas9 to obtain some of the best‐performing variants that had a positive effect on PE efficacy. These variants were named PE6e‐g. The latest PE6 series variants provide customized choices for different scenarios in practical applications while maintaining high efficiency.^129^ In practical applications, PE6a can be utilized to minimize the size of the gene‐editing system. Alternatively, PE6c and PE6d can be employed when prioritizing the twinPE scheme or performing edits that necessitate highly structured RT templates. On the other hand, PEmax∆RNaseH^130^ and PE6b can be employed for edits that do not require highly structured RT templates, aiming to minimize insertions or deletions. Irrespective of the chosen scheme, the evolved and modified Cas9 structural domains of PE6e‐g can further enhance the PE efficiency of specific loci.

In 2024, Gao's team^131^ developed the circular RNA‐mediated prime editor (CPE) system. This system is based on CRISPR‐Cas12a and circular RNA, showing a preference for T‐rich genomic regions and offering the potential for multigene editing. Adamson's team^132^ identified the small RNA‐binding protein La as a key regulator of PE efficiency. By combining the N‐terminal structural domain of the La protein with the PEmax system, they created the innovative PE7 system,^132^ which significantly enhanced the editing efficiency of gene loci associated with diseases.

PE offers greater possibilities for gene manipulation due to its precision, versatility, and flexibility. Compared with previous gene editing techniques, PE excels in processing large fragments of DNA and handling more precise or complex mutation scenarios. However, it is crucial to acknowledge that PE is also more complex to use, despite the availability of numerous auxiliary design tools.^133^, ^134^, ^135^ Users still need to invest significant effort in utilizing PE. Additionally, gene therapy based on PE currently heavily relies on the carrying capacity and delivery efficiency of delivery vector technology. While existing studies have shown the potential of PE, it is important to note that its effectiveness and safety in humans have yet to be fully proven.

### 2.6 Other systems

In addition to the well‐known Cas9 system, other types of gene editing systems also contribute significantly to the field of gene therapy. Cas12a and Cas12f are equally important DNA gene editing tools.

In 2015, Zhang's research team^139^ reported a novel gene editing system called CRISPR/Cas12a (previously known as CRISPR/Cpf1) that differed from Cas9. They demonstrated that the Cas12a protein does not require TracrRNA and can recognize T‐rich PAM sequences, leading to cross‐cutting at the 5′ end of the sequence and forming sticky ends. In 2018, Doudna's research team^140^ observed that Cas12a exhibits target‐activated, nonspecific single‐stranded deoxyribonuclease cleavage activity. They combined Cas12a with isothermal amplification to create a method called DNA end‐to‐end targeted CRISPR transport reporter, which enables rapid and reliable detection of DNA. Compared with Cas9, the crRNA of Cas12a is significantly shorter than the gRNA of Cas9, making it easier to target multiple sites simultaneously. Additionally, Cas12a can identify A/T‐rich regions in the genome, expanding the application potential of CRISPR gene editing. These advantages have attracted significant attention, as indicated in Table 4. Furthermore, numerous new detection systems^141^, ^142^ and gene editing systems^131^, ^143^ based on Cas12a are continuously being developed.

**TABLE 4:** The progress of the clustered regularly interspaced short palindromic repeat/CRISPR‐associated genes (CRISPR/Cas)12a and the CRISPR/Cas12f systems.

Name	Mutation(s)	PAM	Size	References
AsCas12a		TTTV	1307	^139^
LbCas12a		TTTV	1228	^139^
AsCas12a	S542R/K607R	TYCV	1307	^148^
AsCas12a	S542R/K548V/N552R	TYCV	1307	^148^
LbCas12a	G532R/K595R	TWTV	1228	^148^
LbCas12a	G532R/K538V/Y542R	TATV	1228	^148^
FnCas12a		TTN	1346	^149^
enAsCas12a	E174R/S542R/K548R	TTTV	1307	^150^
enAsCas12a‐HF1	N282A	TTTV	1307	^150^
FnCas12a TEXT	T5 ssDNA exonuclease was fused to the N terminal of FnCas12a	NTTV	1607	^151^
cCas12a	The 5′ terminal modified crRNA was covalently ligated to a site‐specifically modified Cas12a.	TTTN	1307	^143^
AsCas12a Ultra	M537R/F870L	TTTV	1307	^152^
eaFnCas12a		YTV	1346	^153^
AsCas12f1		NTTR	422	^146^
CasMINI	Derived from the type V‐F Cas12f(Cas14)system	TTTR	529	^154^
Un1Cas12f1	Redesigned the natural guide RNA of Un1Cas12f1	TTTR	529	^155^
SpaCas12f1		NTTY	497	^156^
enAsCas12f		NTTR	422	^157^
AsCas12f1‐v5.1	N70Q/K103R/A104R/S118A/D364R	NTTR	422	^158^
enOsCas12f1		TTN	433	^147^
enRhCas12f1		CCD	415	^147^
CnCas12f1		NCCD	497	^159^
AsCas12f‐YHAM	F48Y/S188H/V232A/E316M	NTTR	422	^160^
AsCas12f‐HKRA	I123H/D195K/D208R/V232A	NTTR	422	^160^

Abbreviation: PAM, proto‐spacer adjacent motif.

In 2018, Doudna's team^144^ reported the discovery of a compact RNA‐guided nuclease family called CRISPR/Cas12f (also known as Cas14), which consists of approximately 400–700 amino acids. This nuclease was found to have the ability to target single‐stranded DNA (ssDNA) cleavage without strict sequence requirements. In 2020, Siksnys laboratory^145^ further identified a series of ultra‐mini Cas12f nucleases with 5′ T‐ or C‐rich PAM‐dependent double‐stranded DNA cleavage activity. This discovery, as shown in Table 4, has since attracted the attention of numerous researchers. In 2021, a team led by Ji^146^ developed AsCas12f1, consisting of only 422 amino acids. In 2023, Yang's team^147^ developed enOsCas12f1 (433 amino acids) and enRhCas12f1 (415 amino acids). These breakthroughs open up possibilities for all‐in‐one adeno‐associated virus (AAV) packaging. Previously, the primary obstacle to distributing in vivo gene‐editing systems was the loading limit of a single AAV.

When discussing small gene editing systems, it is important to mention the IscB and TnpB proteins encoded by prokaryotic transposons, which are believed to be the predecessors of Cas9 and Cas12 nucleases, respectively.^161^, ^162^ In 2021, Zhang's team^163^ reconstructed the evolutionary origin of the CRISPR/Cas9 system and discovered three highly abundant but previously uncharacterized programmable RNA‐guided nucleases, namely IscB, IsrB, and TnpB. Subsequently, the Siksnys team^164^ confirmed that TnpB can cleave DNA near the 5′ end of the TTGAT transposon‐associated motif (TAM) through guidance from the right element RNA (reRNA). In 2023, Wang's research team^165^ developed a method for large‐scale exploration of a novel TnpB gene editor, which further demonstrated the functionality of TnpB in transposon expansion. They successfully screened and obtained new miniature and highly active TnpB editors, namely ISAam1 (369 amino acids) and ISYmu1 (382 amino acids), in human cell lines.^165^ Also in the same year, Hui's team^166^ extensively optimized and modified the IscB system through RNA structure optimization, protein modification engineering, and other technologies. They successfully obtained an efficient editing variant of IscB (enIscB).^166^ These studies have advanced the miniaturization of gene editing systems and contributed to the application and development of in vivo gene editing therapy.

In addition to the various DNA gene editing systems mentioned earlier, RNA gene editing is also noteworthy. In 2016, Zhang's team^167^, ^168^ elucidated the first novel CRISPR system targeting RNA, known as Cas13a (previously referred to as C2c2). This system is guided by a single crRNA and can be programmed to specifically reduce the expression of certain mRNA. Subsequently, Doudna's team^169^ further uncovered the mechanism by which Cas13a generates mature crRNA and carries out RNA cleavage. Later, Cas13b, 13c,^170^ and 13d^171^ were successively identified. Furthermore, the CRISPR/Cas13 system has been extensively employed for the diagnosis and treatment of viral infections,^172^, ^173^ as well as for RNA‐targeted labeling of live cells.^174^ Cas13 has shown great potential for gene therapy due to its ability to cut single‐stranded RNA. In a study, the researcher utilized CasRx to target pathogenic tau pre‐mRNA and reduce the fraction of dysregulated tau isomers in a neuronal model of frontotemporal dementia.^171^ Another study conducted by Yang and colleagues demonstrated that Cas13 when coupled with AAV, had a significant therapeutic effect on liver diseases^175^ and eye diseases^176^ in mice.

Mitochondrial gene editing is another significant area of research, with researchers achieving success in base editing of mitochondrial genes C to T and A to G.^177^, ^178^ In the past, manipulation of mitochondrial DNA (mtDNA) was limited to targeted destruction of the mitochondrial genome by RNA‐free programmable nucleases.^179^, ^180^ In 2020, Liu and Mougous’ team^177^ developed a mitochondrial cytosine base editor (DdCBE) based on TALE and the double‐stranded DNA deaminase DddA. This base editor catalyzes the C>T transition in human mtDNA. It is worth noting that the DdCBE only exhibits high editing efficiency for 5′‐TC. In 2022, Kim's team^178^ further enhanced the editing capabilities by fusing DddA with the deoxyadenosine deaminase TadA8e, resulting in the development of the TALEDs system. This system allows for A>G conversion on mtDNA. In 2023, a research team led by Wei^181^ reported the development of a novel mitochondrial single‐base editing tool called mitoBEs. They integrated nickase and deaminase to make the tool independent of the DddA system. They also explored its potential application in gene therapy for Leber hereditary optic neuropathy.

In addition to existing systems, several new promising systems are currently being developed. In 2023, Feng Zhang's team discovered an RNA‐guided DNA‐cutting enzyme called Fanzor in eukaryotes.^182^ It is encoded in transposons in eukaryotic genomes and has homology to TnpB from prokaryotes. This enzyme can edit the human genome after reprogramming, which has provided new opportunities for the development of genome editing tools. Compared with the CRISPR/Cas9 system derived from prokaryotes, the Fanzor system is more compact, making it easier to deliver to cells and tissues, and thus more suitable for gene therapy. In the same year, a team led by Zhang^183^ developed the fast locality‐sensitive hashing‐based clustering (FLSHclust) algorithm, which successfully identified 188 novel CRISPR systems and validated the functionality of four of them. One notable discovery was a candidate type VII CRISPR system, distinct from known types, featuring a small Cas7–Cas5 effector complex and a unique interfering protein with the β‐CASP domain, suggesting potential for RNA editing. In 2024, Liu's team^184^ reported a new gene editing tool, hydrolytic endonucleolytic ribozyme (HYER). It recognizes and cleaves substrates using only RNA molecules, without the need for protein nucleases. Engineered HYER has demonstrated increased specificity and flexibility. These studies pave the way for the advancement of gene editing systems and the potential application of gene therapy.

## 3 APPLICATIONS OF GENE EDITING THERAPY IN CVDs

William Harvey discovered blood circulation in the early 16th century. The first description of CVD in the modern Western world dates back to 1772 when William Heberden made observations on angina pectoris.^185^ In 1938, Carl Muller had already noticed hereditary CVDs.^186^ In 1972, Brown and Goldstein^187^ began research on familial hypercholesterolemia (FH). For their groundbreaking research on low‐density lipoprotein receptor (LDLR) and their role in cholesterol metabolism and control,^187^ they were honored with the Nobel Prize in Physiology or Medicine in 1985. In 1983, the Framingham heart study identified smoking, high cholesterol, hypertension, lack of exercise, and diabetes as significant risk factors for CVD.^188^ In 1986, the X‐linked Duchenne Muscular Dystrophy gene (DMD) was identified through positional cloning.^189^ After that, APOB, PCSK9, and others were successively identified as risk genes for CVDs,^190^ and the research on CVD entered the gene generation.

### 3.1 Targeting genetic mutations associated with CVD

Since their introduction in 1987, statins have become the most commonly used lipid‐lowering drugs for the treatment and prevention of CVD. Statins inhibit cholesterol synthesis by inhibiting hydroxymethylglutaryl coenzyme reductase, thereby reducing lipid levels. However, there are still some patients who are intolerant to statins or do not achieve safe lipid levels even with the highest dose. The efficacy of statins can be affected by mutations in the ApoE gene, while mutations in the SLCO1B1 gene can reduce the liver's ability to uptake statins, leading to increased blood levels and a higher risk of rhabdomyolysis or other diseases.^191^ Similarly, other therapeutic drugs for CVD, such as anticoagulants, antiplatelet agents, and blood pressure‐lowering agents, also face concerns regarding dosage, toxicity, and metabolism. Therefore, there is an urgent need for research and development of safer and more effective drugs for CVD. Targeting genetic mutations associated with CVD could be a more precise therapeutic strategy, as evidenced by the success of current antibody drugs, antisense nucleotide drugs, and siRNA drugs.

PCSK9 is a serine protease primarily secreted by liver cells and plays a crucial role in regulating cholesterol homeostasis. Inhibiting PCSK9 can effectively lower levels of low‐density cholesterol (LDL‐C). Currently, there are four approved drugs targeting PCSK9. These include Repatha (Evolocumab), a monoclonal antibody drug developed by Amgen; Praluent (Alirocumab), another monoclonal antibody drug developed by Sanofi and Regeneron; SINTBILO (Tafolecimab), a monoclonal antibody drug developed by Innovent; and Leqviq (Incisiran), a siRNA drug developed by Novartis.

ANGPTL3, a member of the angiopoietin‐like protein family, plays a crucial role in regulating lipid metabolism. Notable drugs that target ANGPTL3 include Evkeeza (Evinacumab), a monoclonal antibody developed by Regeneron. However, the clinical development of Vupanorsen (AKCEA‐ANGPTL3‐L Rx), an ASO drug codeveloped by Pfizer and Ionis, has been terminated due to its association with dose‐dependent increases in hepatic fat. On a positive note, early results from Arrowhead's siRNA drug ARO‐ANG3 in Phase 1 show promise.^192^ This siRNA treatment specifically targets ANGPTL3 mRNA, demonstrating good tolerability and effectiveness in reducing circulating concentrations of atherogenic lipoproteins.

ApoC3 is a member of the apolipoprotein C family, which can influence the metabolism of plasma triglyceride‐rich lipoproteins. It achieves this by inhibiting the activity of enzymes and receptors such as lipoprotein lipase and hepatic lipase (HL) on the surface of vascular endothelial cells. Currently, the only approved drugs that target ApoC3 are Ionis’ ASO drug Waylivra (Volanesorsen). Additionally, Arrowhead's siRNA drugs ARO‐APOC3 and VSA001, as well as Ionis’ ASO drug Olezarsen, are in Phase III of development.

Lp(a) is a cholesterol‐rich macromolecular lipoprotein composed of apolipoprotein a [apo(a)], encoded by the LPA gene, and apolipoprotein B100 (apoB100), which is linked to it through a disulfide bond. This lipoprotein can deposit on the walls of blood vessels and promote atherosclerosis. Currently, there are no approved drugs specifically targeting Lp(a). However, there are promising drugs in Phase III, such as Novartis’ ASO drug Pelacarcen and Amgen's siRNA drug Olpasiran, which aim to target Lp(a) more safely and effectively.

In addition, the treatment of transthyretin amyloid cardiomyopathy (ATTR‐CM) and DMD is also worth noting. ATTR‐CM is caused by mutations in the transthyretin (TTR) gene, resulting in the deposition of amyloid fibers outside the cells. This condition is characterized by progressive neuropathy and cardiomyopathy. Currently, the only approved treatment for ATTR‐CM is Pfzer's TTR tetramer stabilizer Vyndaqel (Tafamidis). However, several drugs have been used to treat another disease called ATTR multiple neuropathy. These drugs include Alnylam's siRNA drugs Ambattur (Vutrisiran) and Onpattro (Patisiran), Ionis’ ASO drug Tegsedi (Inotersen), and AstraZeneca and Ionis’ jointly developed ASO drug Wainua (Eplunersen). Among these drugs, Ambattur is currently undergoing a Phase III clinical study to evaluate its effectiveness in ATTR‐CM patients.

DMD is a rare inherited X‐chromosome‐linked disease caused by a mutation in the gene encoding dystrophin. Patients with DMD experience inflammatory reactions shortly after birth, leading to muscle fibrosis, atrophy, and degeneration, ultimately resulting in respiratory and/or heart failure. Currently approved drugs for DMD treatment include Sarepta's ASO drugs Exodys 51 (Eteplarisen), Vyondys 53 (Golodirsen), and Amodys 45 (Casimersen), as well as NS Pharma's ASO drug Viltepso (Viltolarsen). These drugs work by mediating exon hopping to generate truncated, yet still functional, anti‐muscular atrophy proteins. Additional approved drugs include PTC Therapeutics’ corticosteroid drug Emflaza (Deflazacort) and the small molecule drug Translarna (Ataluren). Translarna can bind to the termination codon, allowing premature termination of mRNA to be read through and resulting in the production of full‐length functional proteins. Another approved drug is the dissociative steroid Agamine (Vasorone), jointly developed by Santhera and ReveraGen.

### 3.2 Modifying gene expression to reduce risk factors

The understanding of CVDs has expanded with the development of the Human Genome Project. Initially, only single gene factors related to CVDs were known. However, with ongoing research, genome‐wide association studies have identified over 100 gene locations and a substantial number of regulatory elements linked to human vulnerability to CVD.^193^, ^194^ These risk genes are usually common genetic variations with minor effects. Nevertheless, their accumulation increases the risk of CVDs. It is important to note that nongenetic risk factors, such as obesity and diabetes, are also influenced by genes. As research on pathogenic mechanisms advances, a greater number of pathogenic variants and key targets have been uncovered. Modifying these genes through gene editing technology to permanently reduce the risk of CVD in patients is a valuable pursuit. It is worth mentioning that gene editing technology has also played a significant role in identifying and validating these essential genes.^195^

According to population‐based case‐control research, CXCR4 is believed to have a strong association with the risk of coronary heart disease. Runmin et al.^196^ conducted a study using the CRISPR/Cas9 method to knock out the CXCR4 gene and found that this intervention significantly reduced cholesterol outflow, which supports his previous findings. To validate its regulatory function in lipoprotein metabolism, Nagiec and colleagues^197^ utilized the CRISPR/Cas9 system to generate chromosomal breakage at the TRIB1 location in HepG2 cells. Similarly, Lalonde et al.^198^ used the CRISPR/Cas9 technology to remove TNF‐sensitive regulatory regions to assess the influence on the expression of candidate gene AIDA and validate coronary heart disease risk genes. Fang's team^199^ employed CRISPR‐based technology to study the genetic variation that controls the responsiveness of vascular endothelial cells to hemodynamics. To determine the role of the 66 bp genomic region in regulating endothelial cell PLPP3, the team successfully selected CRISPR‐deficient isogenic adult aortic endothelial cell lines.^199^ Erdmann's team^258^ utilized the Cre recombinase method to create a gene knockout mouse model that targeted the risk gene NEXN for dilated cardiomyopathy (DCM). The KO mice displayed dilated ventricular lumen and systolic dysfunction, along with characteristics resembling endomyocardial fibroelastosis (EFE), on the 6th day after birth. Their study highlights the significance of NEXN in both DCM and EFE. The research team led by Cowan^200^ utilized the CRISPR/Cas9 system to specifically target the gene of the orphan G protein‐coupled receptor 146 (GPR146) in a mouse model. Their findings revealed that a defect in the GPR146 gene resulted in a significant reduction in plasma cholesterol levels in both wild‐type and LDLR‐deficient mice.

CVD encompasses a wide range of conditions, with pathogenic mechanisms that can vary across multiple dimensions. This complexity offers diverse perspectives for mitigating the risk of CVD. TLR2's proinflammatory activity was discovered by Yingge Wang through the use of CRISPR/Cas9 gene knockout on human coronary artery endothelial cells.^201^ Hai and Ritchie,^202^ through quantitative trait locus localization and CRISPR/Cas9 gene editing, revealed that SOAT1 exon 2 deletion and ACAT1 N‐terminal truncation play crucial roles in cholesterol metabolism in macrophages. They provide ideas for CVD treatment from an inflammatory, cholesterol metabolism perspective. Additionally, Castellani et al.^203^ utilized the CRISPR/Cas9 system to disrupt the mtDNA replication regulatory factor TFAM. The finding revealed that this disruption resulted in alterations in site‐specific nuclear DNA (nDNA) methylation, differential expression of particular genes, and modifications in cell signal transduction. These changes ultimately had an impact on human cardiovascular health.^203^ Tarling and her team^204^ employed the CRISPR/Cas9 system to establish an in vivo model for investigating the loss of RNF130 function. Their research provided evidence that RNF130 functions as an E3 Ubiquitin ligase, specifically ubiquitinating LDLR. This ubiquitination process leads to the redistribution of LDLR away from the plasma membrane. The study further confirmed that RNF130 serves as a novel posttranslational regulator, influencing both the abundance and activity of liver LDLR. These findings hold significant implications for targeting the LDLR pathway as a means to reduce plasma LDL‐C levels.

Furthermore, the pathogenic risk posed by genetic variants in terms of drug‐induced cardiotoxicity is equally noteworthy. Cardiotoxicity is a severe adverse drug reaction, and certain genetic variations may be a crucial contributing factor in certain cases. Doxorubicin is a chemotherapy drug belonging to the anthracycline class, which has shown effectiveness in treating various malignant tumors. However, it is important to note that doxorubicin can cause cardiac toxicity in many patients. In a study conducted by the Brunham team,^205^ they utilized the CRISPR/Cas9 system to mediate the destruction of TOP2B in human pluripotent stem cell‐derived cardiomyocytes (hPSC‐CMs). The results of this study revealed that the knockout of this specific topoisomerase significantly reduces the sensitivity of hPSC‐CMs to doxorubicin‐induced double‐stranded DNA breakage and cell death. These findings indicate the potential role of TOP2B in the pathogenesis of cardiotoxicity. Also of note, Bär and his colleagues^206^ employed AAV‐mediated gene therapy to induce the expression of telomerase in both in vitro and in vivo models of doxorubicin‐induced cardiotoxicity. The increased levels of telomerase aid in preserving mitochondrial function and safeguarding the heart against the detrimental effects of doxorubicin‐induced cell apoptosis.

### 3.3 Potential for personalized medicine

Advances in genetic variant testing have not only uncovered numerous CVD risk factors and therapeutic targets but have also enabled early detection of CVD. This early detection allows for timely interventions in potential patients to mitigate their risk of developing the disease. Furthermore, for diagnosed patients, it also enhances patients’ understanding of their condition and enables the customization of personalized healthcare plans.^207^ The development of gene editing techniques has further expanded the horizons of research and therapy.

Human illnesses are complex and progress slowly. However, researching humans is limited by factors such as time, space, ethics, and techniques. In many cases, researchers can only gather information about a disease when patients actively seek medical attention. However, by the time patients come forward, the disease has typically progressed to a certain stage, resulting in missed data on the early stages of the disease process. Additionally, the course of the disease is often influenced by various factors, including the patients’ different living environments, life experiences, and habits, which might hinder an accurate determination of the true cause of the disease. Cellular and animal models based on human cases offer valuable tools for research and personalized medicine. These models enable the reduction of external variables, allowing for more accurate and focused studies. They also offer a suitable platform for personalized therapies, particularly gene editing therapies.

For patients with hereditary mutations, gene‐specific repair represents the most direct treatment approach. Mechanistic studies utilizing cellular and animal models, tailored gene therapy protocols, and comprehensive evaluation of treatment effectiveness at various stages can serve as a comprehensive framework for the advancement of personalized gene therapy. Despite existing challenges and limitations that require further attention, the potential of gene editing therapies for personalized CVD treatment is promising.

## 4 CHALLENGES AND LIMITATIONS OF GENE EDITING THERAPY IN CVDs

### 4.1 Off‐target effects

Researchers have consistently expressed concerns about the potential off‐target consequences of gene editing.^208^ Ensuring the safety of gene editing procedures remains a top priority in this field and a strong motivation for the upgrading of gene editing technologies. From protein‐based targeting with ZFN and TALENs to RNA‐based targeting with CRISPR/Cas systems, all are aimed at ensuring the safety of gene editing.

The development of high‐fidelity Cas9 variants aims to minimize nonspecific DNA binding and cleavage activity, thereby reducing unexpected shearing events.^209^ Screening smaller, more active, and safer deaminases has also helped address the unpredictable off‐target effects caused by deaminases that use their ssDNA and RNA binding ability to transport Cas9 proteins.^210^ Additionally, the development of inhibitors for Cas9 and deaminases provides the opportunity to precisely control the activity of gene editing systems.^211^, ^212^ By reducing the editing window, base editing precision can be improved and bystander mutations caused by the same bases in the target region can be reduced.^213^

Previously, the CRISPR/Cas system was commonly referred to as the “scissors of genes.” However, in human cells, a new gene editing tool called dCas9‐SSAP has been successful in precisely editing long sequences without causing any DNA breaks.^214^ A team is currently working on developing safeguard single‐guide RNA to reduce the risk of off‐target effects while enhancing the effectiveness of the treatment.^215^ Despite the continuous advancements in technology, maintaining vigilance remains crucial.

### 4.2 Delivery methods

The current limitations of gene therapy primarily revolve around the methods used to deliver gene editing systems. Delivery mechanisms for gene editing systems mainly involve physical techniques, viral vectors, and nonviral vectors.^216^, ^217^, ^218^ Achieving precise delivery of a gene editing system to targeted tissue, organ, or cell without eliciting a rejection response in the body is crucial for the success of gene therapy.

The primary limitation of the current delivery system is its limited payload. Recent studies have suggested that advancements in tiny Cas research could enhance the delivery and treatment efficiency of the CRISPR/Cas system.^146^, ^154^, ^155^ The miABE and miCBE,^166^ which are based on the enIscB system, along with the Sdd6‐CBE^210^ developed using AlphaFold2, have demonstrated extensive application value. Furthermore, the advancement of delivery systems capable of accommodating various types and sizes of payloads will also contribute to unlocking the full potential of gene‐edited drugs.

Additionally, safety is a key concern, as delivery systems must minimize immune responses and cytotoxicity while effectively and specifically delivering gene editing systems to various tissues affected by the disease. Fully humanized carriers, like engineered PNMA2 particles,^219^ could offer greater safety benefits compared with the heterologous delivery systems that are currently widely used. Small extracellular vesicles derived from specific cell types are also anticipated to be effective vectors for delivering gene editing drugs.^220^ Furthermore, DNA nanomachine delivery systems that can be degraded and metabolized in the human body and have good biocompatibility have also made great progress.^221^ The resulting development of drug delivery systems based on chemical modification and controlled self‐assembly of nucleic acids is expected to provide new ideas for gene editing therapy.

### 4.3 Ethical considerations

The ethical issue of gene‐editing therapy is slightly more complex. How to define the importance of hereditary diseases, how to allocate research resources for gene therapy, how to judge between gene therapy and improvement, whether subjective genetic interference will affect genetic diversity, whether gene editing therapy will exacerbate social inequality, and so on are a series of issues that need to be discussed transparently and openly. Additionally, there are numerous ethical and safety issues to be considered, and many decisions and regulations to be made by human beings. As we promote technological progress, we should also consider its ethical implications and the way it is utilized in society.

## 5 CURRENT RESEARCH AND CLINICAL TRIALS IN GENE EDITING THERAPY FOR CVDs

After years of scientific research, significant progress has been made in the treatment of CVD. Nevertheless, there are still several challenges that need to be addressed in the field. One such challenge is the intolerance of certain patients toward existing drugs. Additionally, the exorbitant cost of newer drugs poses a significant barrier, rendering them inaccessible to the majority of individuals. Although current drugs have extended efficacy to 10 weeks or even 6 months, they fail to address the patients’ reliance on continuous medication.

Gene editing therapy shows promise in addressing these challenges. A variety of gene editing systems have been utilized to target key CVD genes in various animal models using different delivery methods, all of which have shown promising results. Recent clinical trials have shown significant advancements in multiple gene editing therapeutic strategies for CVD. The preclinical and clinical gene editing therapies for CVD have established a strong basis for the future exploration and development of innovative therapeutic approaches for CVD.

### 5.1 Preclinical trials of the gene editing therapy for CVD

Angptl3, Pcsk9, and Apoc3 are commonly regarded as promising therapeutic targets for ASCVD,^222^, ^223^ as supported by preclinical trials of gene editing therapy for CVD. Table 5 illustrates the developmental history of CVD gene editing therapy, highlighting the potential of gene editing therapies in targeting these challenging areas that cannot be effectively addressed using conventional small molecules or antibodies.

**TABLE 5:** The progress of in vivo gene editing therapy for cardiovascular disease.

Model	Delivery vector	The CRISPR/cas9 system	Efficiency	References
Mouse	Adenovirus	SpCas9	Pcsk9 > 50% Plasma cholesterol 35−40% ↓	^226^
Chimeric liver‐humanized mouse‐bearing human hepatocytes	Adenovirus	SpCas9	Pcsk9 indels ∼50% Blood Pcsk9 average 52%	^227^
Mdx mouse	Adenovirus	SpCas9	Dystrophin in the mdx muscles transduced ∼50%↑	^228^
Mouse	Adenovirus	BE3	Angptl3 median rate 35% Plasma Angptl3 49%↓ Plasma triglycerides 31%↓ Plasma cholesterol 19%↓	^229^
Mouse	AAV	SaCas9	Pcsk9 indel > 40% Serum Pcsk9 ∼95% ↓ Total cholesterol ∼40% ↓	^60^
Mdx mouse	AAV	SpCas9	The integrated density of dystrophin in cardiomyocytes showed dystrophin protein levels: AAV‐IP injection 52.4 ± 14.3% of WT AAV‐RO injection 71.1 ± 21.0% of WT AAV‐IM injection 69.7 ± 19.8% of WT	^233^
Mdx mouse	AAV	SaCas9	Exon 23 was deleted in ∼2% Dystrophin ∼8% of the normal level	^234^
DMD∆Ex44 mouse	Dual AAV	SpCas9	Dystrophin‐positive myofibers Tibialis anterior 40% Triceps 32% Diaphragm and heart 95%	^235^
DMDΔEx51 mouse	Dual AAV	ABEmax	Dystrophin in the AAV injected tibialis anterior muscle 54.0 ± 1.7% ↑	^236^
RYR2 R176Q/+ mouse	AAV	SaCas9	None of the R176Q/+ mice treated developed pacing‐induced ventricular tachycardia (0%, *n* = 6).	^237^
Ldlr^E208X^ mouse	Dual AAV	SpCas9	LDLR Indels ∼25% Correction mutation ∼6.7% Hepatocytes expressed LDLR ∼20%	^238^
Mouse	AAV	SpaCas9	Pcsk9 −1 indel 16.6%	^239^
hPLN‐R14del mouse	AAV	SaCas9	Ventricular tachycardia threshold ∼45%↑	^240^
RBM20^R636S^ mouse	AAV	ABEmax	RBM20 corrected 66%	^241^
Mouse	Lipid nanoparticles	SpCas9	Serum TTR 97%↓	^243^
Mouse	Nanocarrier	SpCas9	Pcsk9 ∼60% Plasma LDL‐C ∼30%↓	^244^
Mouse	lipoMSN	SpCas9	Pcsk9 50%↓ Apoc3 80%↓ Angptl3 85%↓ Serum cholesterol 43.18%↓	^245^
Mouse	Lipid nanoparticles	SpCas9	Angptl3 median rate 38.5% Serum Angptl3 65.2%↓ Serum LDL‐C 56.8%↓ Serum TG 29.4%↓	^246^
hEx45KI‐mdx44 mouse	Lipid nanoparticles	SpCas9	Dystrophy positive fibers 38.5%	^247^
Mouse	eVLPs	ABE8e	Pcsk9 63% Serum Pcsk9 78%↓	^248^
Cynomolgus monkeys	Lipid nanoparticles	ABE8.8	Pcsk9 66% Pcsk9 indel 0.2% Blood Pcsk9 ∼90%↓ Blood LDL‐C ∼60%↓ Blood lipoprotein ∼35%↓	^249^
Cynomolgus macaques	Lipid nanoparticles	ABEmax	Pcsk9 max 34%, average 26% Serum Pcsk9 32%↓ Serum LDL 14%↓	^250^
Cynomolgus monkeys	GalNAc‐lipid nanoparticles	SpCas9	Angptl3 61% Blood Angptl3 89%↓	^251^

*Note*: ↑, Upward arrows indicate an increased level; ↓, downward arrows indicate a decreased level.

Abbreviations: IM injection, intramuscular injection.; IP injection, intraperitoneal injection; RO injection, retro‐orbital injection.

Adenovirus vectors offer several practical advantages in human gene therapy research. These include a wide range of infections, high infection efficiency, strong tolerance to foreign genes, and nonintegration into the genome.^224^ Despite the challenge of immunogenicity, adenovirus‐based vaccines have been successfully utilized.^225^ In 2014, Musunuru's team^226^ utilized adenovirus to generate the CRISPR/Cas system in the liver of mice and specifically targeted Pcsk9. This resulted in a mutation rate of over 50% in the liver, leading to a reduction in plasma Pcsk9 levels, an increase in liver LDLR levels, and a decrease in plasma cholesterol levels by 35–40%. Importantly, no off‐target mutations were observed. In another study conducted by Musunuru's team,^227^ chimeric liver humanized mice with human hepatocytes were utilized. The researchers employed an adenovirus containing the CRISPR/cas9 system to specifically target the human Pcsk9 gene in 2016. In 2016, Han's team^228^ successfully delivered the CRISPR/cas9 system via adenovirus into the gastrocnemius muscle of newborn Mdx mice. They were able to effectively excise a 23‐kb genomic region on the X chromosome that contained the mutant exon 23 in a DMD mouse model. This resulted in the restoration of dystrophin expression at the skeletal muscle sarcolemma. The study successfully demonstrated a high rate of targeted mutations (∼50%), resulting in a significant reduction in the blood level of human Pcsk9 protein. Furthermore, it was confirmed that the occurrence of off‐target mutations was minimal. In 2018, Musunuru's team^229^ utilized the adenovirus packaging CRISPR/Cas9‐BE3 system to target and edit Angptl3 and Pcsk9 in both normal mice and hyperlipidemic LDLR knockout mice. The results showed a median editing rate of 35% with no evidence of off‐target editing.

The AAV vectors are widely acknowledged as versatile vectors for gene therapy due to their wide host range, strong diffusion, long expression cycle, low immunogenicity, and high safety.^230^, ^231^ Various AAV vector platforms have been developed for treating illnesses in different parts of the body, including the central nervous system, eyes, liver, heart, and muscle. However, the AAV vector does face challenges such as limited loading capacity (5 kb), immunogenicity, viral dosage, and other factors. In 2015, Zhang's group^232^ successfully encapsulated SaCas9 and its sgRNA expression cassette into a single AAV vector. They utilized this system to target the cholesterol‐regulating gene Pcsk9 in the liver of mice.^232^ Remarkably, they observed a gene alteration of over 40% within just 1 week of injection, resulting in a significant reduction in both serum Pcsk9 and total cholesterol levels. In 2016, two independent teams, Olson^233^ and Gersbach,^234^ utilized AAVs to deliver the CRISPR/Cas9 system to the Mdx mouse model of DMD. Their objective was to correct gene expression by bypassing mutant dystrophin exons in muscle tissue. Both studies demonstrated a partial recovery of functional dystrophin in myocardial and skeletal muscle fibers, along with improvements in muscle biochemistry and significant enhancement of muscle strength. Following these findings, Olson's team^235^ conducted additional experiments using their complementary AAV delivery system to package CRISPR/Cas9 and a dual AAV delivery system to package ABEmax.^236^ These experiments aimed to perform gene therapy on mouse models of DMD. Autosomal dominant mutations in the ryanodine receptor type 2 (RYR2) gene are often linked to cases of catecholaminergic polymorphic ventricular tachycardia. In 2018, Wehrens and his team^237^ successfully utilized AAV‐delivered CRISPR/Cas9 to specifically disrupt the mutant allele in RyR2 mutant R176Q heterozygous mice in vivo. This study highlights the potential of somatic gene therapy for treating lethal autosomal dominant heart disease. In 2020, Zhou's team^238^ used the CRISPR/Cas9 system delivered by AAV to repair the LDLR gene in the LDLR E208X point mutant mouse strain. This approach partially restored LDLR expression and significantly improved the atherosclerotic phenotype in the LDLR mutant. In 2021, Li's team^239^ utilized the SpaCas9 system packaged with an all‐in‐one AAV to target the Pcsk9 gene in the liver of adult mice. This approach successfully lowered blood cholesterol levels.^239^ A 16.6% indel was observed at the Pcsk9‐1 site in whole liver tissue, while off‐target mutations were minimal (<1%). Mutations in certain regions of the phospholamban (PLN) gene, such as the deletion of Arg 14 (R14 del), are commonly associated with malignant arrhythmias and ventricular dilatation. In 2022, Stilitano and her team^240^ demonstrated improved cardiac function in humanized PLN‐R14 del mice by disrupting the hPLN‐R14 del allele using the AAV‐delivered CRISPR/Cas9 system. Mutations of RNA binding motif protein 20 (RBM20) are strongly associated with familial DCM. In 2022, Olson's research team^241^ utilized AAV to administer the ABEmax system to RBM20 R636Q mutant mice for gene therapy. This treatment successfully restored their cardiac function and extended their lifespan.

Additional delivery mechanisms have been explored in gene therapy for CVD, apart from the commonly used adenovirus and AAV. Especially lipid nanoparticles show great potential. They have low toxicity and immunogenicity, are suitable for different substances, and their particle size and composition are adjustable for optimization.^242^ In 2018, Morrissey's team^243^ successfully demonstrated the effectiveness of in vivo gene editing using lipid nanoparticles to deliver the CRISPR/Cas9 system. Through a single administration, they were able to significantly modify the TTR gene in the mouse liver, resulting in a remarkable decrease of over 97% in serum TTR levels for a minimum of 12 months. In 2019, Jiang's team^244^ utilized nanocarrier‐delivered CRISPR/Cas9 technology to induce loss of function mutations in the Pcsk9 gene in mice. This approach effectively targeted the liver, which exhibited high Pcsk9 expression, resulting in a successful Pcsk9 mutation rate of 60% and a significant 30% reduction in plasma LDL‐C levels. Importantly, no off‐target mutations were observed. In 2020, a team led by Leong^245^ reported the use of liposome‐encapsulated mesoporous silica nanoparticles (lipoMSN) as a successful CRISPR delivery system. This system targeted the genes Pcsk9, Apoc3, and Angptl3 to enhance cardiovascular health.^245^ The team observed a significant reduction in the expression levels of Pcsk9, Apoc3, and Angptl3 by 50, 80, and 85% respectively. Additionally, serum cholesterol levels were reduced by 43.18%. In 2021, Xu et al.^246^ introduced a lipid nanoparticle delivery platform that carries the CRISPR/Cas9 system for in vivo Angptl3 genome editing. This system successfully achieves targeted and efficient knockout of the Angptl3 gene in the liver of mice, leading to significant reductions in serum Angptl3 protein, low‐density lipoprotein cholesterol, and triglyceride levels (reductions of 65.2, 56.8, and 29.4% respectively). In 2021, Hotta's team^247^ focused on delivering the CRISPR/Cas9 system to skeletal muscle using lipid nanoparticles. Their study involved a DMD mouse model with humanized exon sequences, and they achieved the induction of stable genomic exon jumps, effectively restoring the expression of muscular dystrophin in the mouse model.^247^ In 2022, Liu's team^248^ developed the fourth generation of engineered DNA‐free virus‐like particles (eVLPs), which carried the ribonucleoprotein of the base editor. This innovative technique achieved successful editing of 63% of mice liver cells and led to a remarkable 78% decrease in Pcsk9 protein levels in the blood.

Nonhuman primate models have gained significant attention in the field of CVD gene therapy due to their close resemblance to humans. In 2021, Musunuru et al.^249^ conducted a study using lipid nanoparticles to deliver a CRISPR base editor to living cynomolgus monkeys. This approach effectively and precisely altered the Pcsk9 gene. Following a single‐dose therapy, the levels of Pcsk9 and LDL cholesterol in the blood dropped by nearly 90 and 60% respectively, and these reductions were sustained for at least 8 months. Another study conducted by Schwank's team^250^ in the same year utilized a lipid nanoparticle delivery system of the CRISPR/Cas9 ABE system to target Pcsk9 in cynomolgus monkeys. This resulted in up to 34% editing (with an average of 26%) and a decrease of 32% in plasma Pcsk9 levels and 14% in LDL levels. Importantly, no off‐target mutations were detected. In 2023, Bellinger et al.^251^ employed structure‐oriented rational design to enhance GalNAc‐lipid nanoparticles and utilized CRISPR base editing therapy to target the Angptl3 gene in nonhuman primates with LDL receptor deficiency. They successfully achieved 61% liver editing, with minimal editing observed in nontargeted tissues. Following 6 months of administration, the Angptl3 protein in the blood decreased by 89%.

### 5.2 Clinical trials of the gene editing therapy for CVD

In recent clinical trials, gene therapy strategies based on the CRISPR/Cas system have been explored. Verve has developed VERVE‐101, which is considered one of the 11 clinical trials that will shape medicine in 2024.^252^ The trial aims to target liver cells in patients by using lipid nanoparticles to deliver the ABE system. This approach achieves base transformation from A to G at specific sites of the PCSK9 gene, resulting in permanent gene silencing, reduced production of PCSK9 protein, and lower levels of LDL‐C in the blood. By the end of 2023, preliminary human experimental data were released, showing promising results. One study demonstrated a 55% decrease in cholesterol levels after 1 month in a patient who received the maximum dose (0.6 mg/kg) of treatment, and these levels remained relatively low after 6 months. However, there are still concerns regarding the safety of VERVE‐101, as two patients experienced three serious side effects, and one patient's myocardial infarction may be related to the treatment. Apart from VERVE‐101, Verve is also developing other base editing candidate drugs, such as VERVE‐102 targeting PCSK9 and VERVE‐201 targeting ANGPTL3, which are currently being planned to enter clinical trials. NTLA‐2001, developed by Intellia, targets the TTR gene in patients’ hepatocytes using lipid nanoparticles loaded with the CRISPR/Cas9 system.^253^ The goal is to treat ATTR amyloidosis by reducing serum TTR concentrations. Partial results from the Phase I clinical study indicate that patients tolerated the treatment well, as evidenced by safety assessments conducted within the first 28 days after infusion. Patients treated with a dosage of 0.3 mg/kg experienced an average reduction in serum TTR protein concentration of 87% after 28 days. However, the therapeutic effect of NTLA‐2001 on ATTR‐CM has yet to be reported.

Furthermore, in December 2023, the United States Food and Drug Administration awarded Rare Pediatric Disease Drug Designation (RPDD) to HG302, a gene‐edited therapy developed by HuidaGene, for the treatment of DMD. HG302 demonstrates the ability to effectively deliver high‐fidelity Cas12i variants (hfCas12Max) and CRISPR RNA, targeting the splicing donor site in exon 51 of the human DMD gene, directly to muscle cells using a single viral vector. Sarepta and Roche collaborated in the development of Elevidys, a recombinant gene therapy designed for patients with DMD. This therapy involves intravenous injection of AAV virus containing a micro‐dystrophin transgene, which prompts the patient's muscles to produce recombinant proteins with partial dystrophin function. While Elevidys has obtained accelerated approval for marketing, the Phase 3 validation clinical study did not yield favorable results. Therefore, further evaluation of its efficacy is necessary. Notably, in September 2023, a case report showed the unfortunate death of a patient with DMD following high‐dose AAV gene therapy.^254^ It was found that the patient's congenital immune response resulted in acute respiratory distress syndrome. This incident serves as a crucial reminder for us to maintain a high level of vigilance and scrutiny in all aspects of gene therapy.

### 5.3 Future directions and prospects for gene editing in CVD treatment

Moving forward, there are promising possibilities. The evolution of gene editing systems relies heavily on protein engineering, and advancements in the PACE and PANCE systems have allowed for gradual improvements in gene editing tools like the CRISPR/Cas system.^127^, ^128^ Protein engineering plays a crucial role in enhancing the efficiency, size, and safety of gene editing tools. The availability of data and the emergence of artificial intelligence (AI) technologies such as AlphaFold2 and ChatGPT have provided platforms for protein prediction and creation. AI‐based rational design has made it possible to develop efficient and safe gene editing systems that do not exist naturally.

Based on existing research, PE systems demonstrate significant potential. However, as a user, there is an expectation for a more concise and clear usage scheme, which can be achieved through iterative evolution. This can facilitate the development of junior researchers or other related fields. As research progresses, we may witness the emergence of new targeting tools based on specific structures or gene modifications, moving beyond the early protein‐based and current RNA‐based targeting systems. Moreover, the exploration of gene editing systems has expanded from prokaryotes to eukaryotes,^182^ which has opened up possibilities for future development in mammalian and even human gene editing systems. This could potentially address the immunogenicity concerns associated with current gene editing techniques.^255^ Overall, the solid research foundation and the enthusiasm of researchers contribute to a promising future for gene editing technology.

The future of gene therapy requires more careful consideration. Currently, gene therapy heavily relies on advances in delivery methods, which have steadily progressed through the development of protein engineering, AI techniques, and the application of materials science. One potential development is the combination of existing AAV delivery systems, which rely on specific promoters, and protein delivery systems, which rely on specific receptors on the cell surface. This combination enables the creation of targeted modified viruses or completely artificial virus‐like vectors. Additionally, incorporating human‐derived materials into these designs may alleviate concerns about the safety of the gene editing treatment.

Despite the advantages of existing gene therapy programs as one‐time treatments, their high cost remains a barrier for some patients. However, the modularity advantage of gene therapy programs, such as the CRISPR/Cas system, will allow for a significant reduction in the cost of designing future gene therapies. As clinical evaluation systems stabilize and gene therapy protocols become more widely adopted, personalized gene therapy protocols tailored to individuals are expected to become accessible at affordable prices.

## 6 CONCLUSION AND DISCUSSION

**FIGURE 2:**
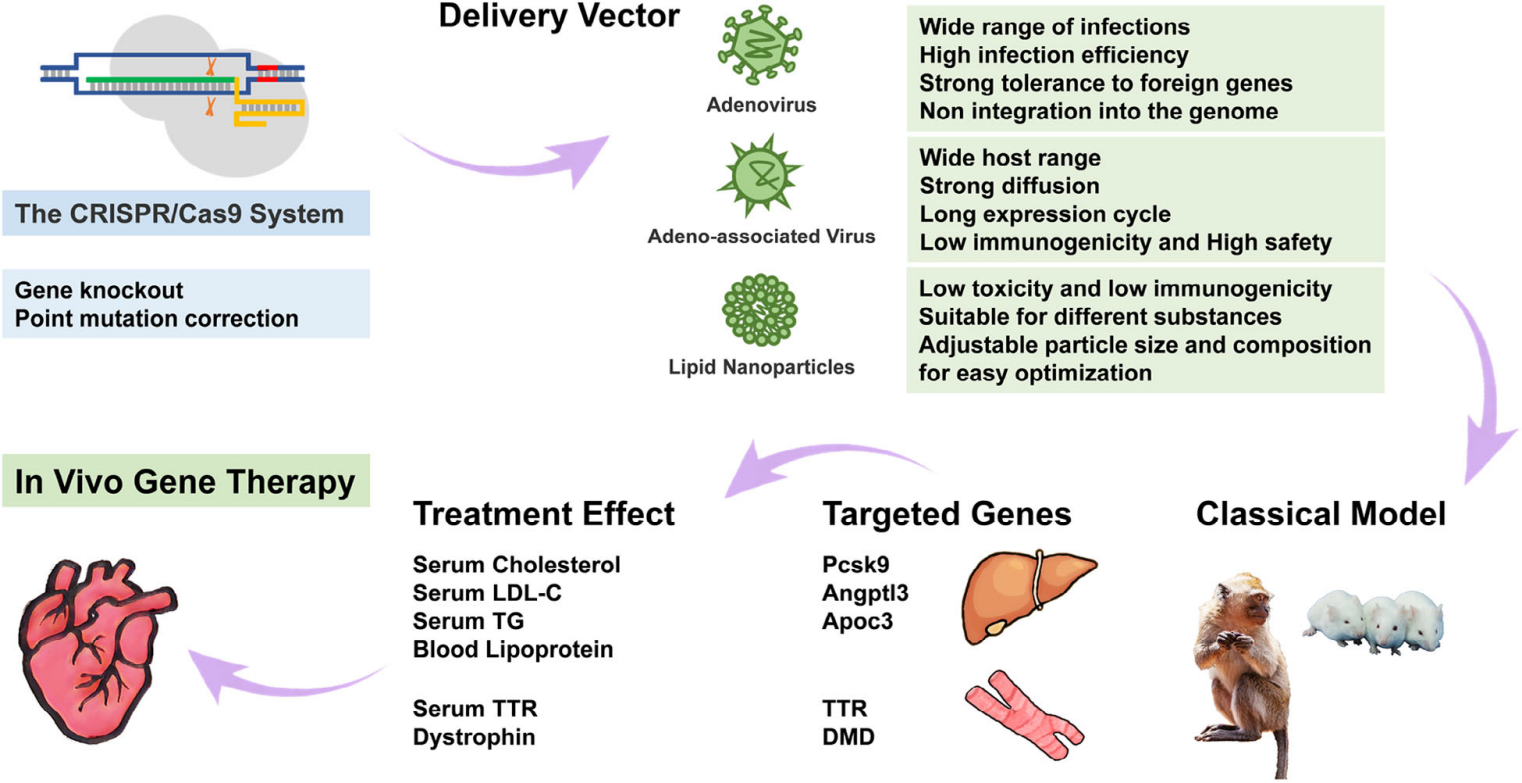
The application of the CRISPR/Cas system for gene editing therapy in CVD. A variety of gene editing systems centered on the CRISPR/Cas system have demonstrated effective gene editing outcomes in various animal models targeting key genes of CVDs with different delivery methods, and furthermore have achieved initial successes in gene editing therapies for CVDs in human clinics. Abbreviations: DMD, dystrophin; LDL‐C, low‐density lipoprotein cholesterol; TG, triglyceride; TTR, transthyretin.

In this review, we provide an overview of the current progress in gene editing tool development and summarize their application in various scenarios based on users’ needs. The challenges and limitations of existing gene editing therapies are also discussed, along with a summary of the ongoing research and clinical trials for CVD. Bases are the fundamental components of carbon‐based life. The ability of the gene editing tools to locate, cut, and modify these bases has proven beneficial in enhancing our well‐being and preventing diseases. In the field of cardiovascular medicine, CRISPR/Cas‐mediated research has shown promising results in vivo gene therapy, as shown in Figure 2. Numerous therapeutic targets have been screened and confirmed to be effective.

The use of gene editing tools in patients with CVD is transforming their lives, as discussed in this review. The combination of the gene editing system with the AAV delivery system has shown significant advancements in the field of CVD. Furthermore, the use of lipid nanoparticles as a delivery method shows great promise. Gene editing therapy offers a sustainable solution for patients, reducing their dependence on ongoing medication. Additionally, it holds promise for individuals with genetic diseases to pursue alternative lifestyles.

However, it is crucial to acknowledge the potential risks associated with gene therapy. A study has indicated that gene therapy for Hemophilia type A may increase the risk of liver cancer in a specific group of patients over a prolonged treatment period.^256^ This has led us to recognize that potential effects on patients that are long term and/or not easily observable after gene therapy are equally worthy of attention. It is important to emphasize that finding a complete cure for these diseases is a complex task that requires meticulous attention to various details, such as effectiveness, durability, and most importantly, safety in terms of avoiding risks like hepatotoxicity, cancer, and immunity concerns, among others. The gene editing system serves as a valuable tool for exploring new possibilities and turning them into reality. There are still unexplored territories that lie between current achievements and the ethical boundaries that must be cautiously navigated. Therefore, the responsible use of gene editing technology is essential for safeguarding and optimizing our lives.

## AUTHOR CONTRIBUTIONS

Xinyu Wu searched the literature and drafted the manuscript. Jie Yang revised and edited the manuscript. Jiayao Zhang examined the literature. Yuning Song designed the review and made a critical revision. All authors agree to be accountable for all aspects of work ensuring integrity and accuracy.

## CONFLICT OF INTEREST STATEMENT

The authors have declared that no conflict of interest exists.

## ETHICS STATEMENT

No ethical approval was required for this study.

## Data Availability

Not applicable.
